# A Fate Map of the Murine Pancreas Buds Reveals a Multipotent Ventral Foregut Organ Progenitor

**DOI:** 10.1371/journal.pone.0040707

**Published:** 2012-07-17

**Authors:** Jesse R. Angelo, Mara-Isel Guerrero-Zayas, Kimberly D. Tremblay

**Affiliations:** Department of Veterinary and Animal Sciences, University of Massachusetts, Amherst, Massachusetts, United States of America; Ecole Normale Supérieure de Lyon, France

## Abstract

The definitive endoderm is the embryonic germ layer that gives rise to the budding endodermal organs including the thyroid, lung, liver and pancreas as well as the remainder of the gut tube. DiI fate mapping and whole embryo culture were used to determine the endodermal origin of the 9.5 days *post coitum* (dpc) dorsal and ventral pancreas buds. Our results demonstrate that the progenitors of each bud occupy distinct endodermal territories. Dorsal bud progenitors are located in the medial endoderm overlying somites 2–4 between the 2 and 11 somite stage (SS). The endoderm forming the ventral pancreas bud is found in 2 distinct regions. One territory originates from the left and right lateral endoderm caudal to the anterior intestinal portal by the 6 SS and the second domain is derived from the ventral midline of the endoderm lip (VMEL). Unlike the laterally located ventral foregut progenitors, the VMEL population harbors a multipotent progenitor that contributes to the thyroid bud, the rostral cap of the liver bud, ventral midline of the liver bud and the midline of the ventral pancreas bud in a temporally restricted manner. This data suggests that the midline of the 9.5 dpc thyroid, liver and ventral pancreas buds originates from the same progenitor population, demonstrating a developmental link between all three ventral foregut buds. Taken together, these data define the location of the dorsal and ventral pancreas progenitors in the prespecified endodermal sheet and should lead to insights into the inductive events required for pancreas specification.

## Introduction

The embryonic endoderm initiates as an epithelial sheet that covers the ventral surface of the developing embryo from approximately 7.0 days *post coitum* (dpc) until the late headfold stage (∼8.0 dpc). By the 2 somite stage (SS, ∼8.25 dpc) the anterior-most endoderm and underlying tissue has created a slight depression resulting in the production of the anterior intestinal portal (AIP) [1]. The creation of this fold heralds the onset of gut tube formation and distinguishes the dorsal and ventral surface of the developing gut tube. Foregut tube formation occurs in a rostral to caudal manner and by 9.5 dpc (∼25 SS) the foregut has extended completely and initiated organ budding, producing the thyroid bud, liver bud, dorsal pancreas bud and ventral pancreas bud. Between 9.5–9.75 dpc the lung buds appear and the emergence of the foregut organ buds is complete [2].

Transplant and explant studies in mouse and chick have demonstrated that the onset of endodermal organogenesis requires secreted factors from adjacent mesodermal tissues [3], [4], [5]. Genetic studies, mainly from lower vertebrates have implicated Wnt, fibroblast growth factor, retinoic acid and the bone morphogenetic protein pathways as being essential for the initiation of endoderm organogenesis, however such studies detailing the role for particular members of these signaling pathways in the mouse have generally been confounded by their multiple essential roles throughout development [6]. Furthermore, despite the medical importance of the ventral foregut organs, little is know about the organ precursors prior to their overt specification. An understanding of the location of the endodermal organ progenitor populations prior to the onset of budding will allow for the better design of experiments aimed at determining the molecular events required for the initiation of organogenesis.

Fate mapping the foregut organ precursors not only allows for the identification of the organ progenitor populations prior to their differentiation but can also provide information regarding morphogenetic movements of the organ precursors. For example, previous fate mapping experiments demonstrated that the liver bud arises from 2 distinct endodermal cell populations [7]. Symmetric left and right lateral populations give rise to the bulk of the liver bud while the ventral midline of the endoderm lip (VMEL) cells produce descendants confined to the ventral midline of the developing liver bud. These results, in coordination with another fate mapping study performed with slightly earlier embryos, have allowed for a better understanding of gut-tube closure in the mouse [7], [8].

One goal of our work is to identify the pancreas progenitors in the endodermal sheet prior to their molecular specification. To perform these studies we DiI labeled small regions of the endoderm prior to specification, allowed the marked embryos to develop through the pancreatic budding stage and then used an antibody against pancreatic and duodenal homeobox 1 (PDX1) to determine if the dorsal and ventral pancreas buds co-localize with the dye labeled endoderm. Between the 10–11 SS PDX1 expression is localized to the emerging dorsal and ventral pancreas buds [9], [10]. Although PDX1 specifically demarcates the pancreas buds until 9.5 dpc, by 10.5–11.5 dpc PDX1 is also detected in non-pancreatic tissues including the caudal stomach endoderm, bile duct and in the rostral duodenum [11], [12], [13].

In the following manuscript, DiI labeling is performed on 2–11 SS embryos that are cultured until ∼9.5 dpc. Using these methods we find that the dorsal pancreas precursors arise from the endoderm that overlies somites 2–4 throughout the early somite stages examined (2–11 SS). The majority of ventral pancreas precursors are located in the left and right endoderm caudal to the lateral liver precursors and adjacent to the first somite at the 6 SS. Between the 9–11 SS the ventral pancreas precursors are found in the lateral regions of the ventral foregut lip. Finally, we find that VMEL cells not only produce descendants in the gut tube and the liver bud as described previously [7], but also contribute to much of the thyroid primordium and to the midline of the ventral pancreas bud in 9.5 dpc embryos. Taken together these results provide information on the location and organization of the foregut organ precursor population prior to and coincident with the onset of organ specification.

## Materials and Methods

### Ethics Statement

All animal studies were approved by the Institutional Animal Care and Use Committee, University of Massachusetts, Amherst protocol #2009-046.

### Embryo Culture and DiI Labeling

CD-1 (Charles River) females were mated with CD-1 studs and the morning of the copulation plug defined as 0.5 dpc. To obtain embryos between the 2–11 SS, females were routinely sacrificed early on day 8 of development. After removal from the uterus, embryos were immediately and quickly dissected in warmed and equilibrated (at least one hour in a 5% O_2_, 5% CO_2_, 37°C incubator) dissection media [DM: 10% Fetal bovine serum, 90% DMEM (Lonza, 12-709)] under a Nikon SMZ1500 dissection microscope equipped with a 37°C stage. After dissection, embryos were held in a 4-well dish (Nunc) in a 5% O_2_, 5% CO_2_, 37°C incubator in culture media [CM: 75% rat serum (Valley Biomedical, AS3061), 25% DMEM (Lonza, 12-614) supplemented with Pen/Strep (Gibco), non-essential amino acids (Lonza) and GlutaMAX (Gibco)]. Embryos were individually labeled with Chloromethylbenzamido-DiI (CM-DiI, Molecular Probes) as described previously [7], except that the DiI was used at a concentration of 0.02–0.04 µg/µl in 0.3M sucrose. The position of the labeled cells was recorded manually and documented using epifluorescence and bright field images produced using a MicroPublisher 5.0 RTV camera and Qimaging software. After manipulation, each labeled embryo was temporarily incubated in a single well of a 4-well dish, as noted above, until all embryo manipulations were complete. For long-term culture, embryos were placed into individual glass bottles with 1–1.5 ml of 37°C equilibrated CM and placed into roller culture as described [7]. Bottles were re-gassed at least once during the ∼30 hour culture period. At the end of culture the extraembryonic tissues were removed, the quality of each embryo noted, the somites counted and then embryos fixed in 4% paraformaldehyde overnight (O/N) at 4°C. Embryos were then washed 2X in phosphate-buffered saline (PBS) and imaged to document the position of the labeled cell descendants and quality of the embryo. After imaging, the embryos were dehydrated, xylene treated and mounted in paraffin wax for sectioning.

### Fate Map Construction

**Figure 1:**
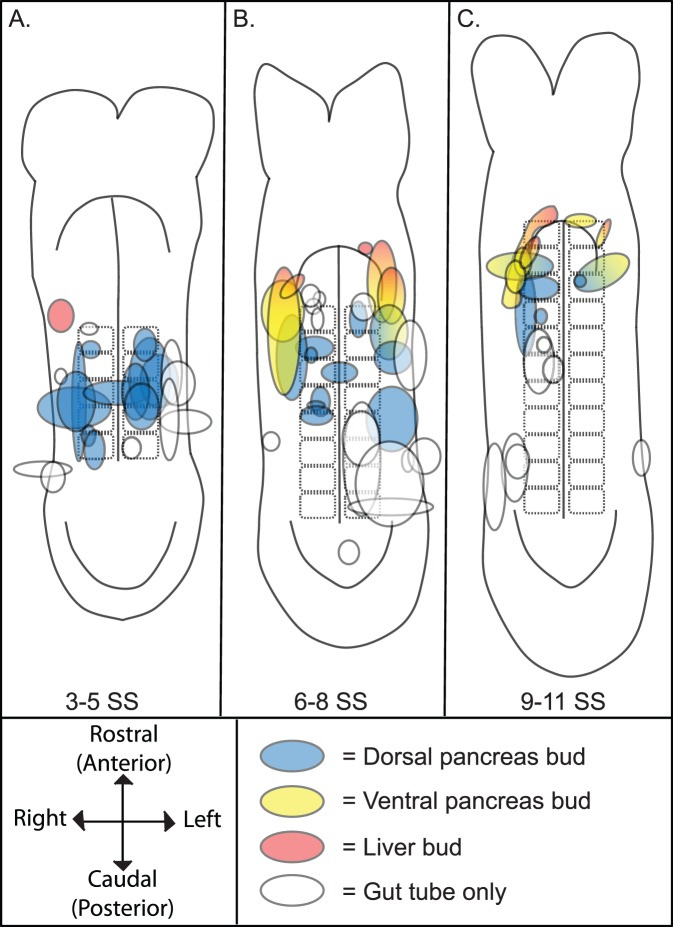
Fate map of the 3–11 SS caudal foregut endoderm. **A–C.** Fate maps summarizing the results obtained from DiI labeling the endoderm of individual 3–5 SS (A) 6–8 SS (B) and 9–11 SS (C) embryos and culturing through ∼9.5 dpc. Each shape represents the position and size of the DiI labeled endoderm from a single embryo at the stage indicated by each map. The color indicates which organ bud those labeled cells contributed to at the end of culture. DiI labeled cells that contributed to the dorsal pancreas bud are colored blue, those that contributed to the ventral pancreas bud are yellow, contributors to the liver bud are red while groups of cells which gave rise solely to gut tube are in white. Because multiple embryos were often labeled in similar domains, the color of each shape is transparent and thus darker shades of a color indicate overlapping labeled endoderm that gave rise to the same bud. Labeled groups of cells that gave rise to 2 organ buds are bi-colored and each color placed in the presumptive half that gave rise to the indicated organ. For ease of view, all the data points that gave rise to gut tube alone were placed on top of any colored shapes. A simple compass indicating the embryonic axis is provided. The orientation of the axis is preserved in all fate map cartoons (Figures 2,3,5 and Figure S1).

Because portions of the region to be fate mapped are found in the curved region of the “U” shaped embryo, the flat rostral foregut fate maps were created by photographing appropriately staged embryos in whole mount and acquiring frontal, lateral and posterior images at the same magnification. Images from a single embryo were merged and the easily distinguishable surface features such as headfolds, AIP, caudal intestinal portal (CIP) and somites traced with a drawing tool to create the 2-dimensional embryo cartoons (Figure 1A–C). Given that the endoderm to be examined overlies and is proximal to the somites, the shape and organization of which are extremely regular, the labeled endoderm could easily be recognized and recorded in relation to these and other identifiable structures such as the AIP.

### Immunofluorescence

Each embryo used in this analysis was sectioned at 7 µm and all sections collected on 1 or 2 slides. Slides were dewaxed in xylene and rehydrated through an ethanol series. Antigen retrieval was performed by microwave treating for 10′ in Tris buffer (0.01 M Tris base, pH 10). Slides were then cooled for 1 hr at room temperature (RT), washed with PBS with 0.1% Tween-20 (PBT), blocked with 0.5% milk in PBT at RT for 2 hours and hybridized with primary antibody (in 0.05% milk/PBT) O/N at 4°C. After three PBT washes the sections were treated with secondary antibody (Molecular Probes; 1∶500) at RT for 1 hour. After washing 2X in PBS for 30 minutes, nuclei were visualized with 4′,6-diamidino-2-phenylindole dihydrochloride (DAPI, Molecular Probes, 1∶10,000) for 4 minutes and coverslipped with ProLong Gold Antifade Reagent (Invitrogen). Sections were imaged on either a Nikon Eclipse TE2000-S inverted microscope with a Retiga EXi Fast camera or a Nikon Eclipse Ti inverted microscope with an Andor DR-228C camera. Both microscopes use NIS Elements imaging software. Primary antibodies used were: rabbit anti-PDX1 (1∶2000, Abcam, ab47267), guinea pig anti-PDX1 (1∶1000, Abcam, ab47308) and mouse anti-TTF1 (Thermo Scientific, MS-699-R7). PDX1 immunofluorescnce (IF) was performed on each labeled embryo to distinguish the dorsal and ventral pancreas buds. To gain confidence in the identification of the thyroid bud, TTF1 IF was performed on similarly staged wild-type embryos as well as on a limited number of VMEL labeled embryos (data not shown). The liver bud was identified by its characteristic morphology.

## Results

We previously created a fate map of the early embryonic endoderm anterior to the first somite using DiI [7]. To ascertain the location of the pancreas progenitors in the current study we used a similar strategy, but instead labeled the endoderm posterior to the previously identified liver progenitor domain. The results described herein were produced by manually labeling individual early somite embryos with DiI, recording the position of the labeled tissue and analyzing the labeled cell descendants 28–30 hours later. During the culture period, the prespecified endoderm becomes internalized via the process of gut tube formation and foregut organogenesis is initiated. A combination of immunohistochemical and morphological criteria were used to assess the location of the labeled cells at the end of the culture period.

### Creation of a Caudal Foregut Fate Map

To assess the location of the pancreatic progenitors prior to the onset of pancreas-specific gene expression, the bulk of the fate mapping experiments were performed on 3–11 SS embryos. Because of the dramatic morphological changes that are apparent between the 3 and the 11 SS, and since short developmental windows within this period have successfully been used to follow the progression of organ precursors [7], the data is grouped into fate maps representing 3–5, 6–8 and 9–11 SS embryos (Figure 1). The fate maps are composed of the following: the 3–5 SS map is comprised of data from 22 different labeled embryos, the 6–8 SS map is generated from 31 labeled embryos and the 9–11 SS map generated from 24 independently labeled embryos.

Each shape on the map represents the data acquired from a single labeled embryo. Since the size, shape and position of the labeled endoderm at the onset of culture differed, these attributes were approximated by the placement, size and shape of each data point in the map. If the labeled group of cells contributed to a budding endodermal organ, then that data point was allocated a color: yellow for ventral pancreas bud contribution, red for liver bud contribution and blue for dorsal pancreas bud contribution. If a labeled group of precursors contributed to more than one organ, the shape was filled in with a gradient of the corresponding colors (labeled descendants were not found in more than 2 organ buds). Finally, if the labeled cells lacked contribution to a budding organ and contributed solely to the gut tube, then the shape representing those labeled cells was filled in white. Since the data points sometimes overlapped, each shape and its color were made partially transparent. As a result, areas on the fate map with the darkest shade of any one particular color represent areas where multiple labeling experiments gave rise to the same organ (Figure 1).

As has been observed in other murine endoderm fate mapping experiments as well as herein, coherently labeled groups of precursors give rise to coherent patches of labeled cell descendants [1], [7]. These results indicate that the endodermal sheet extends and grows without widespread cell mixing. If organ fate was attributed to a group of cells, the cell descendants were either found exclusively in that organ, or in that organ and in adjacent regions of the gut tube. Similarly, if two organ fates were attributed to a group of cells, then labeled cell descendants were found in both organs and potentially in a portion of the gut tube spanning those organs. Given this assembly of data in the fate maps, the most accurate method of assessing the location of organ precursors is to attribute organ fate to the area of significant overlap rather than to the region labeled in a single labeling experiment.

### Pancreas Progenitor Identification

The earliest reported onset of endogenous PDX1 is at the 9–10 SS in the lateral endoderm proximal to the closing ventral foregut, presumably demarcating the ventral pancreas [9], [10], [14]. A second PDX1-positive domain located in the dorsal gut appears at the 10–11 SS [9], [14], identifying the dorsal pancreas bud. PDX1 expression can be used to specifically identify the pancreas buds at least until 9.5 dpc. To assess pancreatic fate of the DiI labeled endoderm at 9.5 dpc, serial sections were made and PDX1 IF performed. DiI colocalization with PDX1-positive cells was used to attribute pancreas fate to the labeled progenitor population.

### Dorsal Pancreas Progenitor Identification

**Figure 2:**
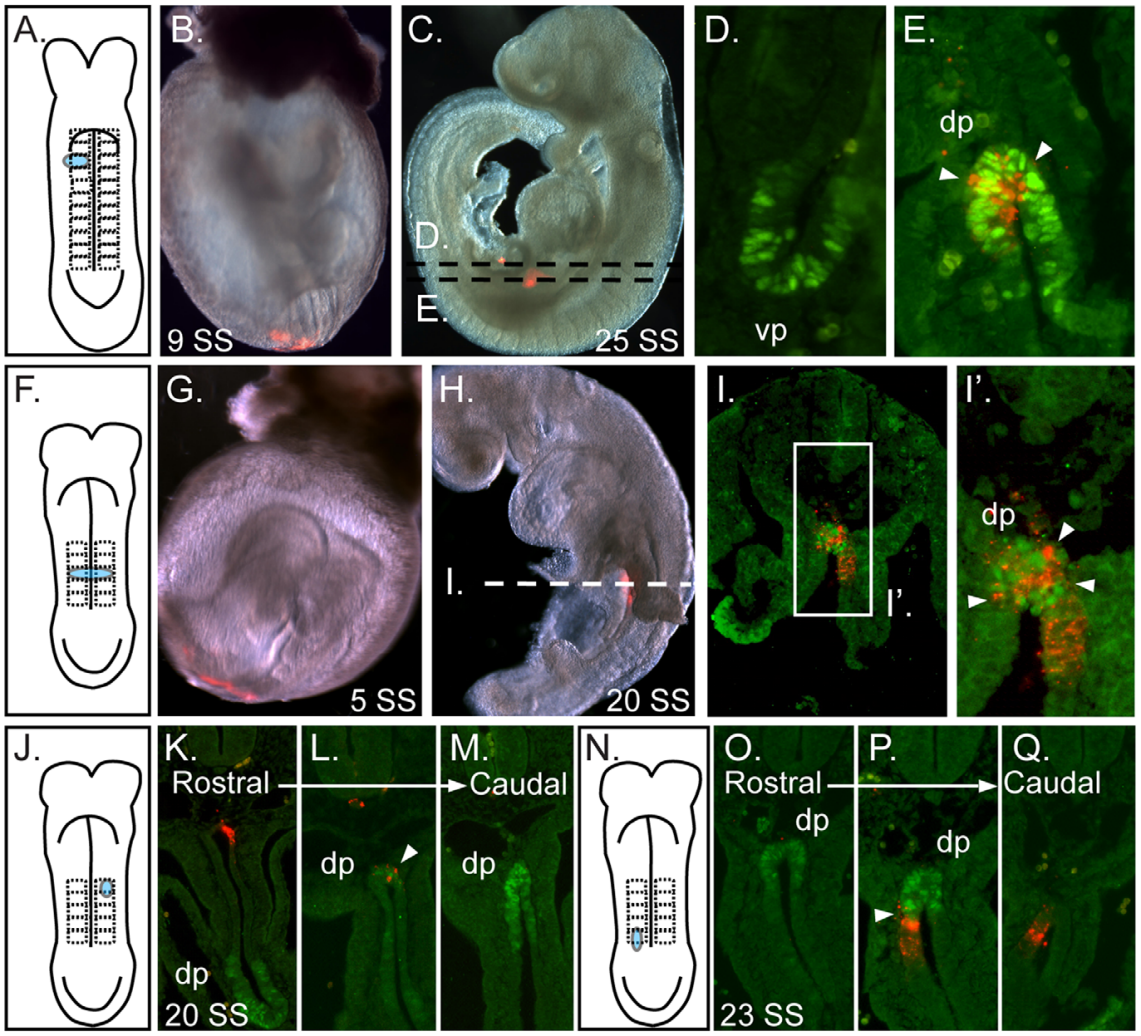
Identification of the dorsal pancreas progenitors. **A–B.**A cartoon depicting a 9–11 SS embryo labeled over the third somite and slightly lateral on the right side (A) and a bright field/fluorescence merged image of the actual 9 SS embryo revealing the DiI (red) labeled cells (B). **C.** After culture, the merged image reveals that the resultant 25 SS embryo contained DiI labeled descendants in the dorsal gut (lower red) as well as a smaller patch of visible descendants in the ventral/lateral gut tube (upper red). **D–E.** Transverse sections as indicated by the dashed lines in (C) revealed the co-localization of DiI (red) in the PDX1-positive (green) dorsal pancreas bud (dp, E) but not in the ventral pancreas bud (vp, D). **F–H.** A cartoon representing a 5 SS embryo labeled over the third somite pair, as indicated in the cartoon (F) and shown in the actual labeled 5 SS embryo (G) that was cultured to the 20 SS (H). **I.** Section analysis of the embryo indicated in (H) with PDX1 (green) reveals DiI labeled descendants throughout the dorsal pancreas (arrowheads in I’, boxed region in I is magnified in I’). **J.** A cartoon of a 5 SS embryo labeled over the left first and second somite, cultured through the 20 SS and processed as above. **K–M.** Transverse sections through this embryo are aligned in a rostral to caudal manner as indicated by the arrow. DiI labeled descendants (red) are found in the dorsal endoderm rostral to the dorsal pancreas (K) and only slightly overlap with the rostral-most portion of the PDX1-positive dorsal pancreas bud (arrowhead in L). All of the more caudal sections of the dorsal pancreas (green) do not contain DiI labeled cells (M). **N.** A cartoon depicting a 5 SS embryo labeled in the endoderm over the caudal most fourth and much of the fifth somite. **O–Q.** Section analysis of the resultant 23 SS embryo, arranged in a rostral to caudal manner as indicated by the arrow, revealed that DiI labeled cells were absent from the rostral regions of the dorsal pancreas bud (green, O) but overlapped slightly with the caudal portion of the dorsal bud (arrowhead, P) and extend into gut tube caudal to the dorsal pancreas bud (red cells in Q). In all sections the arrowheads indicate region of DiI overlap with the dorsal pancreas bud.

Previous fatemapping experiments have demonstrated that the endoderm of the dorsal gut tube arises from the endoderm proximal to the notochord in the unspecified endodermal sheet [7], [8]. Thus it is not surprising that dorsal pancreas colocalization with the DiI labeled cells at the end of culture, indicated by the blue color on the fate map (Figure 1), was restricted to embryos that were labeled on midline-proximal endoderm. Examples of fate mapped embryos that produced descendants in the dorsal pancreas are shown in Figure 2. A single 9 SS embryo labeled with DiI on the endoderm overlying the third right somite, is depicted in cartoon form (Figure 2A) and is directly visualized by the red fluorescence in the fluorescent/bright field merged whole mount view of the actual labeled embryo (Figure 2B). This embryo was cultured for 28 hours, reaching the 25 SS (Figure 2C), and sectioned as indicated. Analysis of all serial sections revealed that while DiI was absent in the PDX1-positive ventral pancreas (Figure 2D), the dye-labeled descendants overlapped with PDX1-positive cells in the dorsal pancreas (Figure 2E). Even a 5 SS embryo, similarly labeled on the endoderm overlying the third somite pair (Figure 2F–G) and cultured through the 20 SS (Figure 2H) produced labeled descendants in the dorsal pancreas (Figure 2I).

Significantly, all labeled embryos that produced descendants in the dorsal pancreas (n = 29, Figure 1A–C) had been labeled in the endoderm overlying somites 2–4 from the 3–11 SS (3–5 SS, n = 12; 6–8 SS, n = 11; 9–11 SS, n = 6, Figure 1) while endoderm labeled exclusively in adjacent regions did not produce dorsal pancreas progenitors (Figure 1). Taken together these data suggest that the endoderm overlying the second through fourth somite pair harbors the dorsal pancreatic progenitors.

Close examination of embryos labeled on and proximal to the boundaries of the dorsal pancreas progenitor domain demonstrates the maintenance of anterior-posterior linearity throughout culture. Dorsal endoderm at the anterior edge of the progenitor domain gives rise to dorsal endoderm both anterior to and within the anterior portion of the dorsal pancreas. Similarly, pre-specified endoderm at the posterior boundary of the dorsal pancreas progenitor domain gives rise to dorsal endoderm located posterior to and on the posterior edge of the dorsal pancreas. For example, when a 5 SS embryo was DiI labeled on the endoderm overlying the first and second somite (Figure 2J), the descendants were found in the non-pancreatic dorsal endoderm immediately anterior to the dorsal pancreas (Figure 2K) as well as a portion of the anterior-most dorsal pancreas (Figure 2L) but were not found in the more caudal portions of the dorsal pancreas (Figure 2M). Similarly when a 5 SS embryo was labeled on the dorsal pancreas boundary and slightly more posterior (right somite 4–5, Figure 2N) the DiI labeled cell descendants were absent from the rostral dorsal pancreas (Figure 2O) but were instead found to slightly overlap with the PDX1-positive domain on caudal edge of the dorsal pancreas (Figure 2P) and to extend into the gut tube posterior to the dorsal pancreas (Figure 2Q).

To extend the temporal analysis of the dorsal pancreas progenitors, the endoderm over the second somite at the 2 SS was labeled. The labeled cells from these early embryos (n = 2) also produced descendants within the dorsal pancreatic endoderm (Figure S1). Taken together these results demonstrate that the dorsal pancreatic bud progenitors overlay somites 2–4 from the 2 SS through the onset of dorsal pancreas specification by the 11 SS.

### Ventral Pancreas Progenitor Identification

From 9.0–9.5 dpc, the ventral pancreas bud is adjacent to the rostrally located liver bud. Our previous fate mapping results suggested that the ventral pancreas progenitors were located at the caudal limit of the liver bud progenitors during the 1–3 SS [7] indicating the maintenance of the relative positions of the 9.5 dpc foregut organs within the prespecified endoderm. Therefore, to identify the location of the ventral pancreas precursors during later SS we focused on the left and right lateral endoderm at the level of, and posterior to, the first somite. Using such a strategy we did not recover any embryos demonstrating significant DiI labeling in the PDX1-positive ventral pancreas bud when labeled from 3–5 SS (Figure 1A, n = 12).

**Figure 3:**
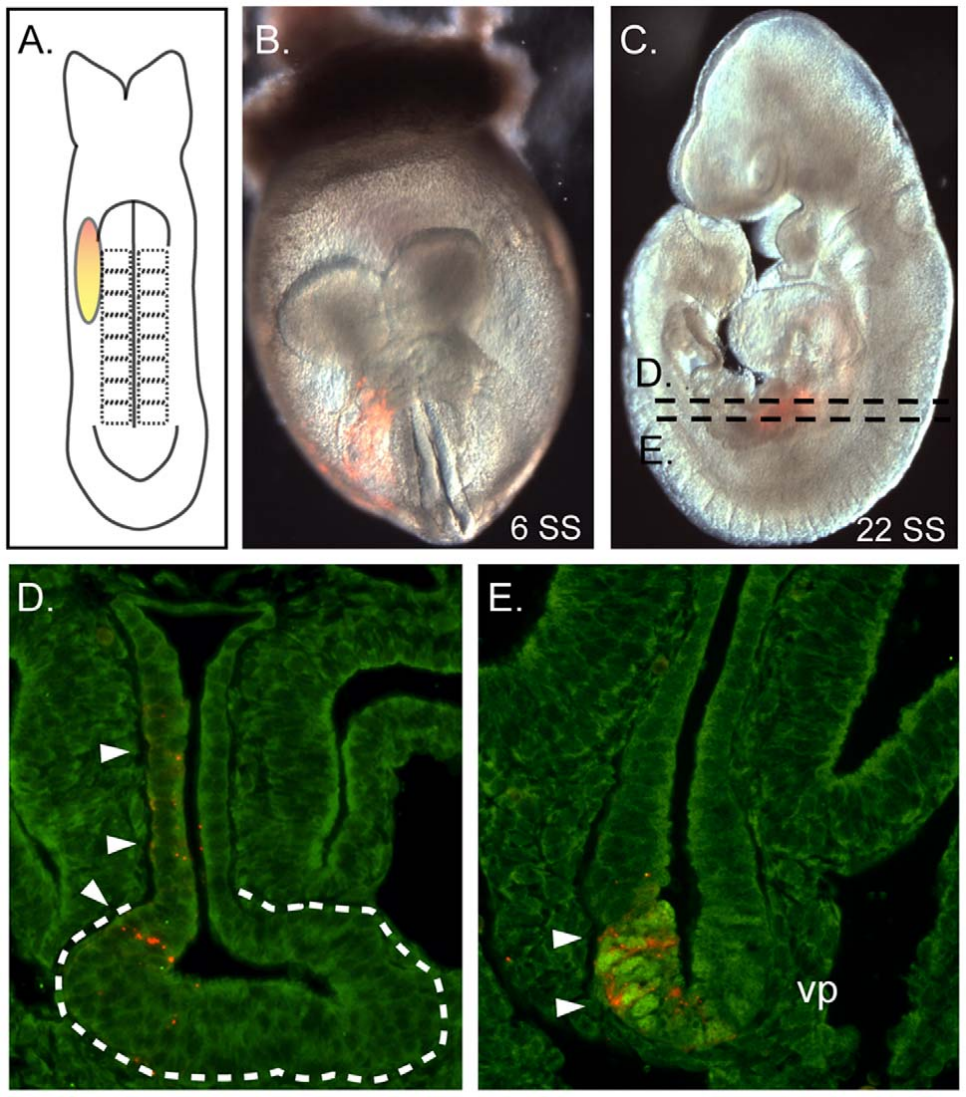
Identification of ventral pancreas precursors. **A–C.**A 6 SS embryo labeled in the lateral endoderm stretching from the right rostral edge of the AIP to the fourth somite, is depicted in the cartoon (A) and in a merged fluorescent/bright field view of the actual labeled embryo at the onset (red cells in B) and at the end of culture (C, 22 SS). **D–E.** DiI labeled cells (red, arrowheads) contribute to the liver bud (outlined by the dashed line) and adjacent gut tube (D) as well as in the lateral ventral pancreas (E, arrowheads) denoted by the prominent nuclear PDX1 immunofluorescence (green).

Ventral pancreas progenitors were successfully and reproducibly labeled in the lateral endoderm, rostral to the edge of the AIP, between the 6–8 SS (Figure 1B, n = 6). For example, the 6 SS embryo depicted in Figure 3 was labeled in the lateral endoderm extending from the right edge of the AIP to the endoderm lateral to the fourth somite (Figure 3A–B). As predicted from the previously published anterior foregut fate map [7], this label produced descendants in the lateral portion of the liver bud (Figure 3D). Other descendants, presumably from a more caudal region of the initial label, were found within the lateral portion of the ventral pancreas bud (Figure 3E). While only a single labeled embryo produced descendants that were found exclusively in the ventral pancreas bud, all other labeled endoderm that produced descendants in the ventral pancreas also contributed descendants to adjacent tissue buds (Figure 1B; n = 4 ventral pancreas and liver bud; n = 1 ventral pancreas and dorsal pancreas bud). At the 9–11 SS, 5 of 8 the labels that produced descendants in the ventral pancreas bud also contributed to other tissue buds (Figure 1C, n = 3 in ventral pancreas and liver bud; n = 2 in ventral and dorsal pancreas). All 9–11 SS embryos that produced descendants in the ventral pancreas bud were found within the left and right sides of the ventral lip of the AIP. Because all DiI labeled cells that were found in the ventral pancreas were confined to the side of the bud corresponding to the side of the initial label, we next sought to determine the location of the endodermal precursors that give rise to the middle of the ventral pancreas bud.

### VMEL Cells Give Rise to the Midline of Multiple Organs

We previously demonstrated that VMEL cells produce descendants in the midline of the anterior gut tube as well as to the midline of the liver [7]. We sought to determine if the ventral pancreas is also derived from this cell population by carefully labeling small regions of the endoderm (∼5–30 cells; data not shown) at the VMEL position with DiI during the 2–8 SS and culturing for ∼30 hours. At the end of culture we examined the VMEL contribution to all ventral foregut organs that are readily identifiable in the 9.25–9.5 dpc embryo including the thyroid, liver and ventral pancreas buds.

**Figure 4:**
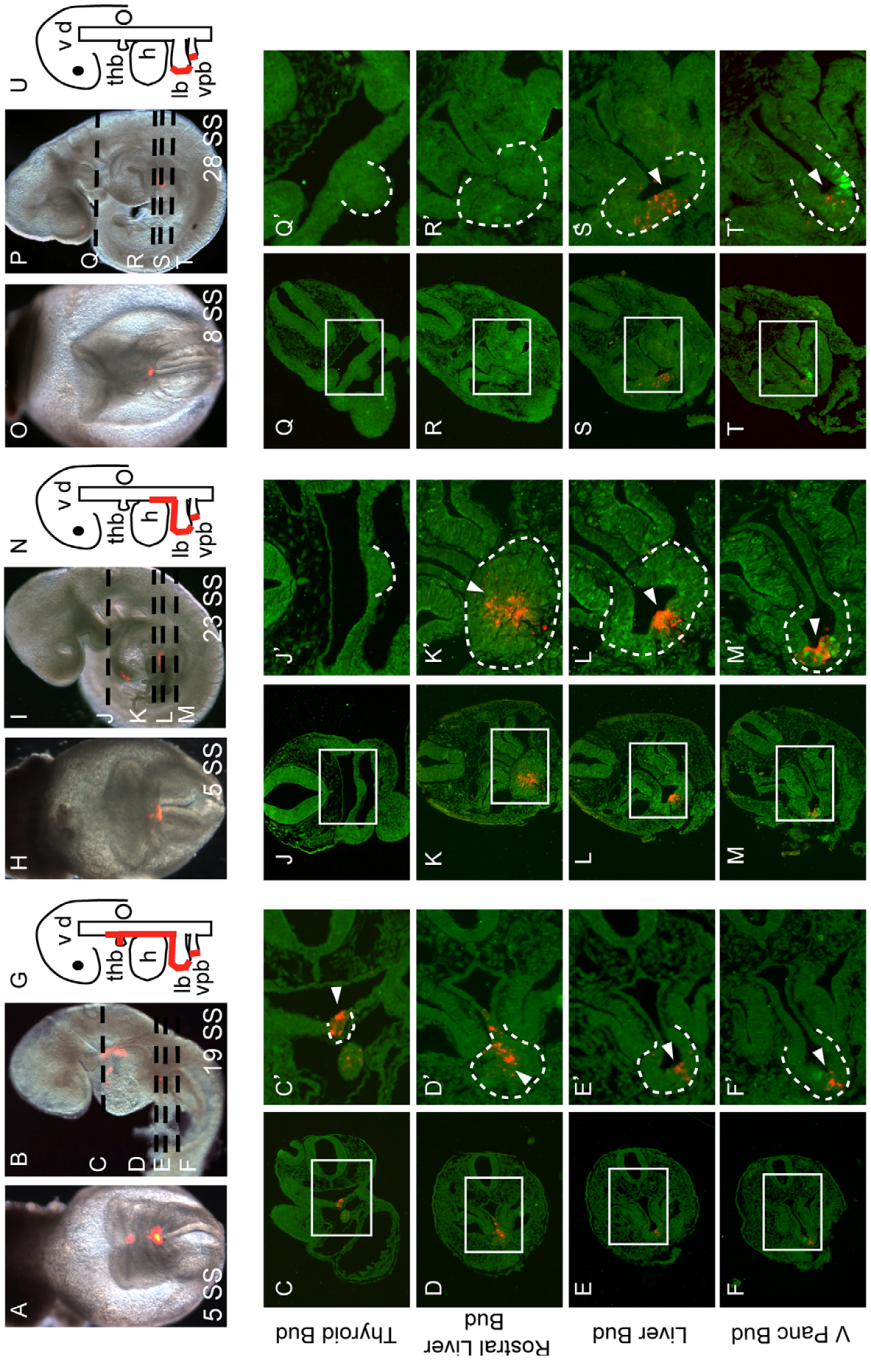
VMEL contribution to multiple foregut organ buds occurs in a temporally restricted manner. **A–B, H–I, O–P.** Bright field/fluorescent merged views of embryos labeled with DiI (red) in the VMEL at the onset (A, H, O) and after ∼30 hours of culture (B, I, P). All embryos were sectioned as indicated (dashed lines in B, I and P) and immunofluorescence performed with PDX1 (green) to identify the ventral pancreas bud. Thyroid buds (C, J, Q) and different portions of the liver bud (rostral liver in D, K, R and liver bud in E, L, S) were identified based on morphology. All sections are presented at low magnification (C–F, J–M, Q–T) and a portion of this field (indicated by the boxed area) presented at high magnification (C’–F’, J’–M’, Q’–T’) where the indicated tissue bud has been outlined by a dotted line and arrowheads used to point to DiI labeled cells within the indicated tissue bud. **G, N, U.** Cartoons summarizing the VMEL contribution (red line) to the thyroid bud (thb), liver bud (lb) and ventral pancreas bud (vpb) for the embryo to the left of the cartoon. **A–G.** This 5 SS VMEL labeled embryo (red, A) was cultured through the 19 SS (B). Section analysis of this embryo revealed DiI contribution to the thyroid (C, C’), a swath of cells in the rostral liver (D, D’) and a limited number of cells in the midline of the liver (E, E’) and ventral pancreas buds (F, F’) as summarized in G. **H–N.** The VMEL labeled 5 SS embryo (H) was cultured through the 23 SS (I). Section analysis reveals no VMEL contribution to the thyroid bud (J, J’) but the presence of VMEL descendants in a swath of rostral liver bud (K, K’), in the midline of the liver bud (L, L’) and in the midline of the ventral pancreas bud (M, M’) as summarized (N). **O–U.** This 8 SS embryo (O) was DiI labeled in the VMEL and cultured through the 28 SS (P). Although labeled VMEL descendants were not found in the thyroid (Q, Q’) or rostral liver buds (R, R’) they were found in the midline of the liver (S, S’) and ventral pancreas buds (T, T’) as summarized (U). v = ventral and d = dorsal gut tube, h = heart.

The results obtained from VMEL labeling experiments are summarized in Table 1 and are demonstrated in Figure 4. Embryos labeled in the VMEL between 2–8 SS gave rise to the midline of the ventral pancreas bud at 9.5 dpc (n = 15/18, Table 1, Figure 4F, M, T). As expected [7], we found that the VMEL population also readily contributed to the liver bud (n = 18/18, Table 1 and Figure 4D–E, K–L, S).

**Table 1:** Summary of each VMEL labeled embryo’s contribution to the 9.5 dpc ventral foregut buds.

Somite Stage^a^	Thyroid Bud	Rostral Liver Bud	Liver Bud ML^d^	V Panc Bud ML
**2**	X^b^	X	X	X
**2**	X	X	X	X
**3**	X	X	X	–
**3**	X	X	X	X
**4**	X	X	X	–
**4**	X	X	X	X
**5**	X	X	X	–
**5**	X	X	X	X
**5**	X	X	X	X
**5**	–c	X	X	X
**5**	–	X	X	X
**6**	–	X	X	X
**6**	–	X	X	X
**6**	–	X	X	X
**6**	–	X	X	X
**6**	–	–	X	X
**8**	–	–	X	X
**8**	–	–	X	X

a Somite Stage of embryo at onset of culture.

b An “X” denotes the presence of DiI labeled cells in this structure.

c A “–“ indicates that no DiI labeled cells were found in this structure.

d ML = midline.

Two other interesting results were also observed. The first is that the VMEL appeared to contribute differently to the rostral portion of the liver bud compared with the remainder of the liver bud (compare Figure 4D to 4E and Figure 4K to 4L). Secondly, we found that the VMEL was capable of producing descendants in all 3 of the ventral midline organs including the thyroid, liver and ventral pancreas buds, albeit in a temporally restricted manner. For example, while contribution to all 3 organs was noted in the majority of the 2–4 SS embryos (n = 4/6, Figure 4A–F, Table 1), all 2–4 SS embryos (n = 6) contained DiI labeled cells that contributed to the thyroid bud. Thyroid bud contribution by the VMEL became restricted by the 5 SS (n = 3/5, Table 1) and was no longer noted by the 6 SS (Table 1). Most of the 5–6 SS VMEL labeled embryos that did not produce descendants in the thyroid bud instead produced descendants in both the rostral and midline of the liver bud as well as in the midline of the ventral pancreas bud (n = 4/5, Figure 4H–M, Table 1). A single 6 SS and both 8 SS VMEL labeled embryos produced descendants that were restricted to the midline of the liver and pancreas buds (Figure 4O–T, Table 1). While these data are consistent with and extend on the previous observation that the VMEL contributes to the gut tube and liver bud [7], these results are the first to show that the VMEL consists of an organ progenitor population capable of contributing to the midline of all ventral midline positioned foregut organ primordium at 9.5 dpc.

## Discussion

**Figure 5:**
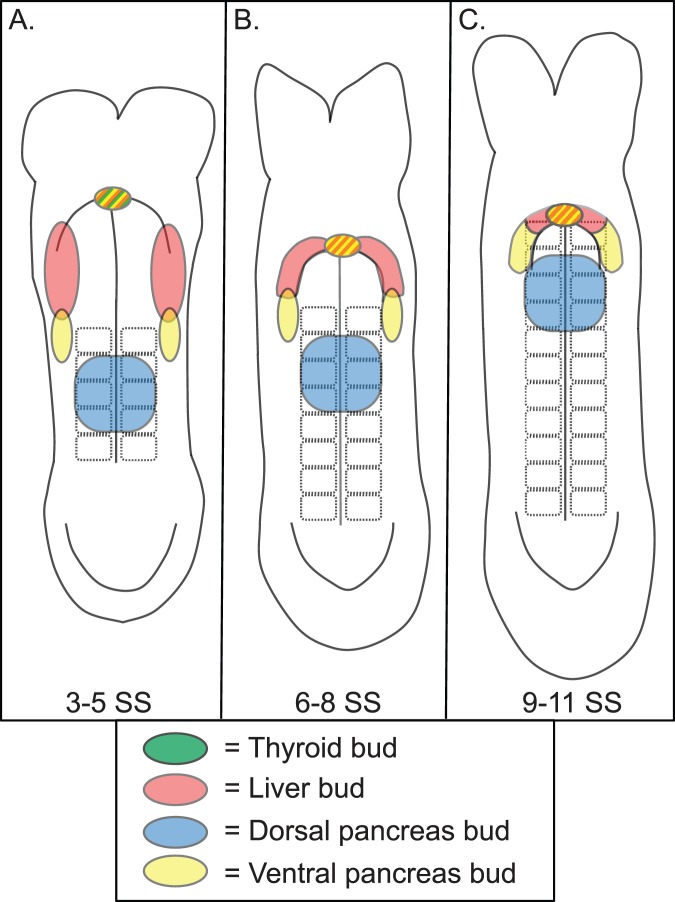
A summary of the location of foregut organ progenitors during early somite stages. **A–C.** Cartoons depicting the position of the foregut organ progenitor populations at the 3–5 SS (A), 6–8 SS (B) and 9–11 SS (C) based on the data presented here and elsewhere [7]. The VMEL contribution in the 9–11 SS embryo has not been directly examined and is assumed. The exposed endodermal progenitor populations that are fated to form the 9.5 dpc organ buds are indicated on each map. Blue indicates a dorsal pancreas bud fate, yellow a ventral pancreas bud fate, red a liver bud fate and green a thyroid bud fate. The absence of thyroid progenitors by the 6 SS and the regression of the lateral liver progenitors by the 9 SS indicate that these progenitors have entered the closing foregut. The VMEL population, indicated by the ventral midline oval, produces midline descendants contributing to the thyroid, liver and ventral pancreas buds in a temporally restricted manner.

As summarized in Figure 5, we found that the dorsal and ventral pancreas precursors exist in identifiable domains within the unspecified caudal foregut endoderm preceding known molecular or morphological hallmarks. Furthermore we present data demonstrating that VMEL endoderm contributes to all three 9.5 dpc ventral foregut organ buds in a temporally restricted manner. At the 2 SS, the VMEL cells are capable of contributing to the thyroid bud, rostral and midline of the liver bud as well as the midline of the ventral pancreas bud. By the 6 SS the VMEL cell contribution to the ventral foregut organs is limited to the rostral and midline of the liver bud and to the midline of the ventral pancreas bud. By the 8 SS, VMEL cell descendants no longer contribute to the thyroid or the rostral portion of the liver bud but are still able to contribute to the midline of the liver and ventral pancreas buds. These results suggest that the VMEL is a multipotent foregut organ progenitor population that contributes to the ventral foregut organs in a temporally defined rostral to caudal manner. While the rostral limit of VMEL contribution to the foregut organ primordium is directly related to the SS at which the VMEL was labeled (Table 1), the caudal limit of VMEL contribution to ventral gut in our culture system is the ventral pancreas bud. *In vivo* lineage tracing will be required to both confirm and expand on the role of the VMEL during later stages of endodermal organogenesis.

### Dorsal and Ventral Pancreas Progenitor Identification

Unlike that described for other vertebrates such as zebrafish [15], [16] and chick [17], the mammalian literature suggests that the dorsal and ventral pancreas buds contribute equal amounts of exocrine and endocrine tissue to the adult organ. Intriguingly, the dorsal and ventral bud each has different molecular requirements for early development suggesting that there are multiple ways to create exocrine and endocrine tissues from the mammalian endoderm [10], [14], [18], [19], [20], [21]. The results presented herein, as well as those recently described elsewhere [22], demonstrate that the dorsal and ventral pancreas buds arise from distinct embryonic endoderm populations, highlighting yet another important difference between these two organ buds. Given that inductive signaling is necessary for many stages of early endoderm organogenesis, an interesting next step will be to delineate the tissues that interact with and presumably induce the dorsal and ventral pancreas buds from the naïve endoderm. Here we have described a potential novel long-term interaction between the dorsal pancreatic progenitor population and mesenchymal tissue proximal to somites 2–4, pointing to these tissues as a potential inductive signaling source.

### A comparison of Pancreas Fate Maps

The location of the dorsal and ventral precursors presented herein, is similar to another recently constructed murine fate map [22] with a few exceptions. For example, while the size and general location of the dorsal pancreas precursors is similar between the 2 fate maps, the precise location relative to somite number is slightly shifted. Here we demonstrate that the dorsal pancreas precursors are located in the endoderm overlying somite 2–4 from 2 SS through the onset of specification while the others locate the dorsal pancreas progenitors to the endoderm overlying somites 3–6 between the 7–9 SS [22].

Although both groups locate the ventral pancreas progenitors in the VMEL and lateral endoderm between the 2–4 SS and throughout much of the ventral AIP by 9S, the two fate maps differ on the location of the ventral pancreas precursors from the 5–9 SS. While Miki *et al* [22], suggest that the ventral pancreas precursors lie in a continuous arc on the ventral AIP lip between 5–8 SS we show that the bulk of the ventral pancreas precursors reside in two non-overlapping domains on the right and left side of the closing AIP. Support for non-continuous placement of the ventral pancreas progenitors in two lateral stripes at these stages is provided by at least three other pieces of evidence: 1) a *Pdx1*-reporter drives *β-galactosidase* expression in two lateral stripes at 7–8 SS [23] 2) detection of the endogenous PDX1 protein in two stripes of lateral endoderm in the 9 SS mouse embryo [9], [10], [14] and 3) the lateral location of the PDX1-positive ventral pancreas progenitors in chick [17].

The differences in the location of the bulk of the dorsal and ventral pancreas precursors in the 2 fate maps could be attributed to inherent morphogenetic differences in the genetic backgrounds used, differences in the extent of PDX1 expression at the endpoints analyzed or the methods used to colocalize PDX1 expressing cells with the DiI labeled descendants. Our analysis was performed at ∼9.5 dpc, when PDX1 is restricted to the dorsal and ventral pancreas buds, while the others analyzed their embryos at 10–10.5 dpc [22], when PDX1 expression is also present in the caudal stomach and rostral duodenum [11], [12], [13], which may result in attributing pancreatic fate to these adjacent gut tissues.

An important difference between the two fate maps includes our observation that the VMEL population contributes to all three ventral foregut organs from the 2–5 SS, while the others observe a more restricted pattern of foregut contribution after VMEL labeling at these early somite stages [22]. Because the VMEL labeling is often faint and, in our hands, most reliably discerned in section analysis the more restricted pattern attributed to the VMEL descendants by Miki *et al* (2012), may simply be due to differences in experimental design, including making the bulk of the DiI observations in whole mount and culturing for longer periods of time, which could result in an inability to detect a faint/dilute DiI signal. It is also possible that the VMEL contribution to the 10.5 dpc gut differs from that of the 9.5 dpc gut. An independent method of VMEL labeling should be used to distinguish these alternatives.

### VMEL Contribution to the Ventral Pancreas Bud

Recent studies of murine *Sox17*, a Sox-related transcription factor, have highlighted the importance of *Sox17* in gall bladder and bile duct formation and have also demonstrated that the 9.5 dpc ventral pancreas bud is actually a composite bud [24], [25]. While the lateral regions of this bud contains pre-pancreatic tissue, the ventral midline of the early ventral pancreas bud is believed to form the gall bladder [24]. These data suggest that the VMEL contributed region of the 9.5 dpc ventral pancreas bud is not only comprised of a progenitor population that is distinct from the lateral ventral pancreas bud cells, but that these progenitors are also functionally distinct: the lateral portions of the bud produce pancreatic tissue while the midline portion of the bud produces the gall bladder.

### Role of the VMEL in Chick, Mouse and Human- a Conserved Progenitor Population

The initial description of a VMEL cell-like contribution to the ventral foregut was described in the chick [26] where two experimental methods, including DiI labeling, were used to reveal that the VMEL cells contributed to the ventral foregut in a rostral to caudal stripe. Although the authors did not examine organ bud contribution of VMEL descendants, they initiated their analysis at early post-gastrulation stages demonstrating that VMEL cells originate from the rostral end of Henson’s node, suggesting that the cells might be acting as a ventral midline organizer.

Regardless of its mechanistic role, given the distinct rostral/caudal ventral midline tract of the VMEL cells in mouse, one hypothesis is that embryos with abnormal VMEL cells would exhibit multiple defects in the ventral midline of endoderm derived organs. Indeed, mice lacking *Hex*, a gene expressed in the extending AIP during early somite stages, including throughout the VMEL region described herein, demonstrate defects in the thyroid, liver and ventral pancreas bud formation [27], [28], [29]. At least some of these phenotypes could be attributed to improper caudal migration of the midline progenitor populations [30].

A survey of the human disease literature finds a devastating pediatric disease, termed Martínez-Frías syndrome, which has been hypothesized to be due to an embryonic ventral midline defect [31], [32]. Patients with Martínez-Frías syndrome have trachea-esophageal fistulas, extra-hepatic (and occasionally intra-hepatic) duct defects, biliary agenesis, occasional annular or hypoplastic pancreas, intestinal fistulas and hypospadias. Many of the phenotypes noted in these patients suggest incomplete closure of the ventral gut midline and are also consistent with defects in the VMEL contribution to the developing gut. Further investigation of the VMEL population at the molecular level is critical as is deciphering the role this population plays in ventral gut morphogenesis.

## Supporting Information

Figure S1 **2 SS embryo contribution to the dorsal pancreas bud. A.** Two 2 SS embryos were labeled with DiI on the endoderm overlying the second somite and cultured through the 18 SS. **B-D.** This 2 SS embryo was labeled in the endoderm overlying somites 1–2 on the left side as indicated (small label in A) and cultured until 18 SS (C). Section analysis of this embryo as indicated (C) demonstrates that the DiI labeled descendants (red) were located anterior to (data not shown) and within the left side of the PDX1-positive (green) dorsal pancreas bud (dp, arrowheads indicate some regions of overlap). **E.** A section through another 2 SS embryo labeled throughout the endoderm overlying the second somite pair as indicated (large label in A). The embryo was cultured through the 18 SS and sectioned to reveal localization of the labeled DiI descendants (red) in the PDX1-positive (green) dorsal pancreas bud (dp, arrowheads indicate some regions of overlap).
(TIF)
