# Supplementary figures and images for: A Fate Map of the Murine Pancreas Buds Reveals a Multipotent Ventral Foregut Organ Progenitor
Source: PLoS One. 2012 Jul 17;7(7):e40707. doi: 10.1371/journal.pone.0040707 (PMC3398925; doi:10.1371/journal.pone.0040707)

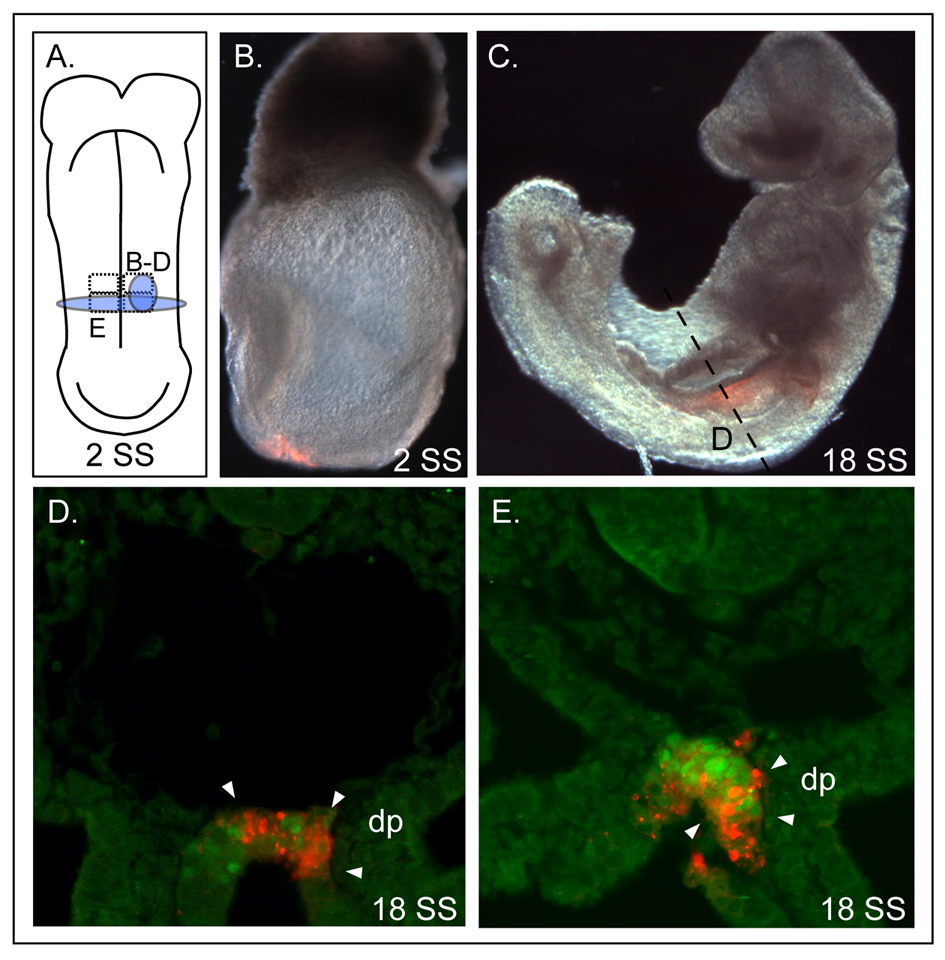

Supplement: Figure S1 — 2 SS embryo contribution to the dorsal pancreas bud. A. Two 2 SS embryos were labeled with DiI on the endoderm overlying the second somite and cultured through the 18 SS. B-D. This 2 SS embryo was labeled in the endoderm overlying somites 1–2 on the left side as indicated (small label in A) and cultured until 18 SS (C). Section analysis of this embryo as indicated (C) demonstrates that the DiI labeled descendants (red) were located anterior to (data not shown) and within the left side of the PDX1-positive (green) dorsal pancreas bud (dp, arrowheads indicate some regions of overlap). E. A section through another 2 SS embryo labeled throughout the endoderm overlying the second somite pair as indicated (large label in A). The embryo was cultured through the 18 SS and sectioned to reveal localization of the labeled DiI descendants (red) in the PDX1-positive (green) dorsal pancreas bud (dp, arrowheads indicate some regions of overlap). (TIF) [file pone.0040707.s001.tif]
